# Case Report: Extranodal marginal zone B-cell lymphoma in the lateral ventricle: literature review

**DOI:** 10.3389/fonc.2026.1733338

**Published:** 2026-05-11

**Authors:** Yimeng Xue, Zhuo Li, Yun Cui, Guilin Li, Qing Chang, Congwei Jia, Jiang Du

**Affiliations:** 1 Department of Neuropathology, Beijing Neurosurgical Institute, Beijing Tiantan Hospital, Capital Medical University, Beijing, China; 2 Department of Pathology, Peking Union Medical College Hospital, Chinese Academy of Medical Sciences and Peking Union Medical College, Beijing, China

**Keywords:** PCNSL, primary central nervous system lymphoma, CNS, Mucosa-associated lymphoid tissue (MALT) lymphoma, lateral ventricle

## Abstract

Primary central nervous system (CNS) mucosa-associated lymphoid tissue (MALT) lymphoma is a rare, low-grade B-cell lymphoma that typically arises in the dura, and its occurrence within the ventricular system is exceedingly rare. We report a case of primary CNS MALT lymphoma located in the right lateral ventricle of a 62-year-old man. The patient underwent gross total resection, followed by postoperative treatment with ifosfamide combined with a Bruton’s tyrosine kinase (BTK) inhibitor, and adjuvant radiotherapy. At the latest follow-up, no evidence of recurrence was observed. A review of the literature revealed only eight previously reported cases of ventricular MALT lymphoma, many of which were initially misdiagnosed on imaging as meningioma, metastasis, or ependymoma. Although chronic inflammation has been implicated in MALT lymphomas outside the CNS, potential infectious or inflammatory triggers in ventricular MALT lymphoma remain unclear. This case expands the recognized anatomical spectrum of CNS MALT lymphoma and proposes a hypothesis that lateral ventricular tumors may originate from the pia mater folds of the choroid plexus, highlighting the need to consider MALT lymphoma in the differential diagnosis of intraventricular lesions.

## Introduction

Primary central nervous system lymphoma (PCNSL) is a rare extranodal non-Hodgkin’s lymphoma characterized by involvement of the central nervous system (CNS) without systemic involvement of the disease. Of PCNSL cases, diffuse large B-cell lymphoma (DLBCL) is the most common tumor type, accounting for approximately 95% (1). In the 2021 World Health Organization (WHO) classification of CNS tumors, mucosa-associated lymphoid tissue (MALT) lymphoma of the dura is a distinctive low-grade lymphoma composed of marginal zone B cells and arising in the dura (2). To date, slightly more than 200 cases of primary CNS MALT lymphoma have been reported (3), with approximately 80% arising from the dura (4). MALT lymphomas can occasionally develop in tissues without mucosa. Some cases arise within the brain parenchyma. Approximately 10.6% of primary CNS lymphomas may involve the ventricular system; however, lesions occurring exclusively within the ventricles are exceedingly rare (5). Previous reports of intraventricular MALT lymphoma are limited to isolated case reports and small case series, and the clinical, radiological, and pathological features have not been systematically summarized. To address this gap, we reviewed all published cases and compiled their key characteristics into a comparative table (**Table 1**) (4, 6–12). MALT lymphomas may be associated with chronic infections (10, 11) and are often radiologically mistaken for meningiomas (9). Overall, these tumors are characterized by slow growth and generally have a favorable prognosis.

**Table 1:** Summary of extranodal marginal zone B-cell lymphoma in the lateral ventricle previously reported.

Case	Year	Age	Sex	Tumor location	Symptoms	Clinical diagnosis	Treatment	Inflammatory condition	Clinical outcome
1 (8)	2005	53	M	Left.LV	Headache, seizure	Meningioma	GTR, adj	None identified	ND at 14 m
2 (9)	2006	63	M	Right.LV	Seizure	Meningioma	GTR, adj	None identified	–
3 (10)	2011	44	F	Right.LV	Headache	–	STR	*Chlamydophila psittaci*	ND at 25 m
4 (7)	2014	65	F	Left.LV	None	Asymmetric choroid glomus	GTR	None identified	–
5 (11)	2022	69	F	Left.LV	Headache	Meningioma	GTR, adj	None identified	ND at 6 m
6 (4)	2022	45	F	Right.LV	None	–	–	Xanthogranulomas	–
7 (12)	2023	48	F	Left.LV	Headache	Ependymoma	GTR, adj	Chronic inflammation	ND at 12 m
8 (6)	2025	52	F	Left.LV	Headache	Meningioma	STR, adj	None identified	–
9	Present report	62	M	Right.LV	Dizziness	Meningioma	GTR, adj	None identified	ND at 6 m

F, female; M, male; Left.LV, left lateral ventricle; Right.LV, right lateral ventricle; GTR, gross total resection; STR, subtotal resection; adj, adjuvant therapy; ND, no evidence of disease; m, month.

In this study, we present a case of primary MALT lymphoma located in the right lateral ventricle in a 62-year-old man. Additionally, we summarized all previously published cases of intraventricular MALT lymphoma, outlining their clinical features and potential pathogenesis.

## Clinical summary

**Figure 1:**
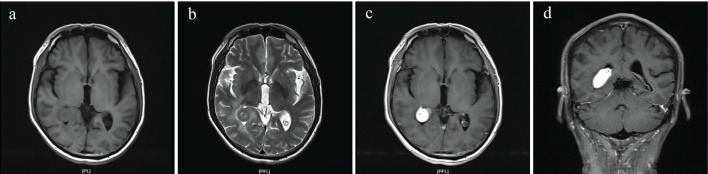
Magnetic resonance imaging (MRI) images showing a mass-like lesion in the right lateral ventricle with isointense signal on T1-weighted imaging (T1WI) **(a)**, mixed high and low signal on T2-weighted imaging (T2WI) **(b)**, and marked contrast enhancement on coronal **(c)** and axial planes **(d)**.

A 62-year-old man presented to our hospital with complaints of dizziness and fatigue for over 1 month. Magnetic resonance imaging (MRI) revealed a mass-like lesion in the right lateral ventricle measuring approximately 17 mm × 22 mm × 31 mm. The lesion was isointense on T1-weighted imaging (T1WI) (**Figure 1a**) and demonstrated heterogeneous high and low signals on T2-weighted imaging (T2WI) (**Figure 1b**). On FLAIR sequences, the lesion appeared isointense to slightly hyperintense. Diffusion-weighted imaging (DWI) showed mild diffusion restriction with corresponding slightly decreased ADC values. Postcontrast imaging demonstrated marked enhancement with well-defined borders (**Figures 1c,d**). The lesion was closely associated with the choroid plexus. Preoperatively, a meningioma or metastatic tumor was considered. Neurological examination revealed that the patient was fully conscious, with normal cranial nerve function, muscle strength, and sensory function. Routine laboratory tests, including complete blood count, liver and kidney function, coagulation profile, and serum electrolytes, were all within normal limits. Cerebrospinal fluid (CSF) analysis showed no evidence of infection or malignant cells. After completing the preoperative evaluation, the patient underwent a right parieto-occipital craniotomy for lesion resection under general anesthesia. Intraoperative findings revealed a grayish-red lesion within the ventricle, encapsulated and solid in nature, with a firm, tough consistency and a rich blood supply. The lesion was closely associated with the choroid plexus. Intraoperatively, the tumor was completely resected. Postoperatively, the patient’s dizziness and fatigue improved, with no new neurological deficits. Follow-up MRI demonstrated complete removal of the lesion and no other intracranial abnormalities.

Of note, other examinations showed no abnormalities. Serological testing for human immunodeficiency virus (HIV), hepatitis B virus (HBV), and hepatitis C virus (HCV) was negative. Lactate dehydrogenase (LDH) and β2-microglobulin levels were within normal limits. Serum immunoglobulin levels and autoantibody profiles were unremarkable. Serum protein electrophoresis showed no evidence of monoclonal gammopathy. Screening for *Helicobacter pylori* was also negative. Bone marrow biopsy revealed no evidence of malignancy. Positron emission tomography (PET) scans were performed to evaluate the whole-body status, with no signs of lymphoma detected elsewhere. Postoperatively, the patient received ifosfamide (1.5 g/m^2^) combined with a Bruton’s tyrosine kinase (BTK) inhibitor (ibrutinib), followed by radiotherapy.

## Pathological findings

**Figure 2:**
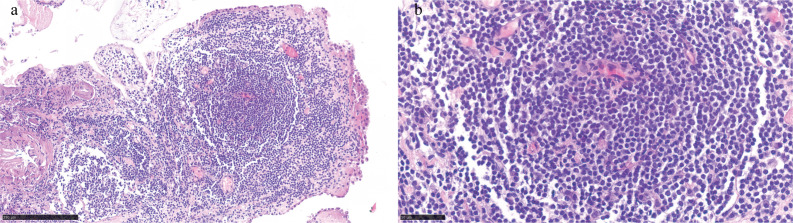
Representative hematoxylin–eosin (H&E) staining images. **(a)** H&E staining revealed marked stromal edema and lymphoid tissue infiltration within the choroid plexus papillae. **(b)** Tumor cells resemble small lymphocytes and monocytoid cells, exhibiting small- to medium-sized nuclei.

**Figure 3:**
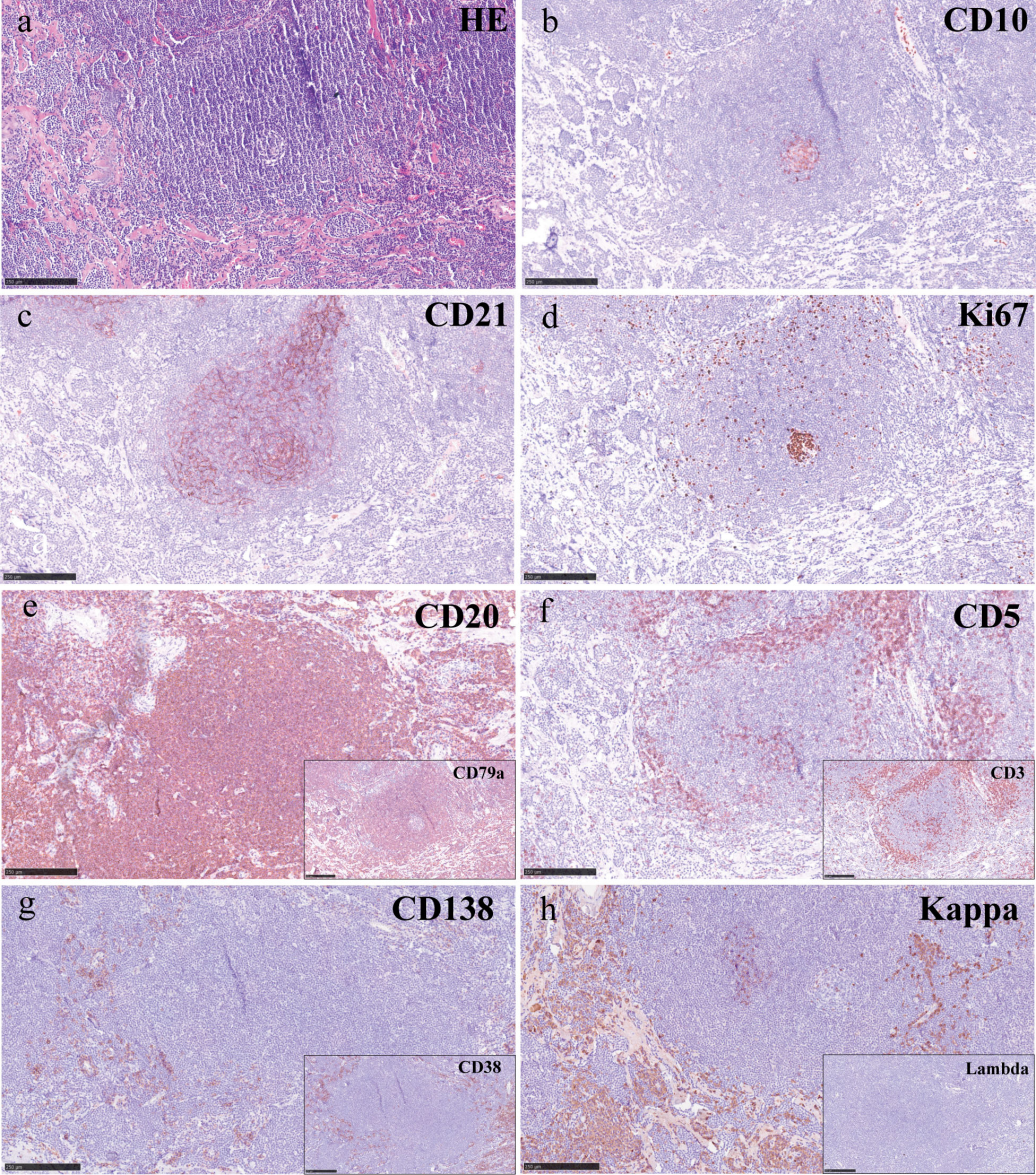
Hematoxylin–eosin staining and immunohistochemical staining. **(a)** H&E staining showed extensive lymphoid tissue proliferation with follicular structure formation. **(b)** CD10-positive cells indicated the presence of residual germinal centers. **(c)** CD21 highlighted the follicular dendritic cell (FDC) meshwork. **(d)** Ki-67 showed high proliferative activity within the germinal centers, while the hyperplastic marginal zone cells exhibited low proliferation (~ 10%). The expanded marginal zone showed positive staining for CD20 and CD79a **(e)**, while tumor cells were negative for CD5 and CD3 **(f)**. **(g)** CD138 and CD38 highlight a subset of tumor cells showing plasmacytic differentiation. **(h)** Light-chain immunostaining demonstrated predominant kappa expression with absent lambda staining, indicating light-chain restriction.

Microscopic examination showed extensive lymphoid tissue proliferation with follicular structure formation within the choroid plexus papillae (**Figure 2a**). Tumor cells resembled small lymphocytes and monocytoid cells, exhibiting small- to medium-sized nuclei (**Figure 2b**). Significant hyperplasia of the marginal zone and partially colonized reactive follicle centers were present. The marginal zone lymphocytes were small, morphologically uniform, and maturely differentiated (**Figure 3a**). In addition, numerous small vascular tufts with collagenization and calcification were observed.

Immunohistochemistry (IHC) staining of CD10- and Bcl-6-positive cells indicated the presence of residual germinal centers (**Figure 3b**). CD35 and CD21 highlighted the follicular dendritic cell (FDC) meshwork (**Figure 3c**). Ki-67 showed high proliferative activity within the germinal centers, while the hyperplastic marginal zone cells exhibited low proliferation (~ 10%) (**Figure 3d**). The expanded marginal zone showed positive staining for the B-cell markers CD79a and CD20 (**Figure 3e**), while CD5, CD3, and cyclin D1 were negative (**Figure 3f**). CD138 and CD38 highlighted a subset of tumor cells showing plasmacytic differentiation (**Figure 3g**). Light-chain immunostaining demonstrated predominant kappa expression with absent lambda staining, indicating light-chain restriction (**Figure 3h**).

Immunoglobulin (Ig) gene rearrangement testing detected clonal rearrangements of *IgH* and *IgK* genes. Based on these findings, the final pathological diagnosis was extranodal marginal zone lymphoma of mucosa-associated lymphoid tissue.

## Literature search strategy

A systematic literature search was performed in PubMed, Web of Science, and Embase databases from inception to January 2026. The following search terms were used in combination: “MALT lymphoma”, “mucosa-associated lymphoid tissue lymphoma”, “central nervous system”, “intraventricular”, “ventricle”, and “choroid plexus”.

Inclusion criteria were as follows: (1) primary CNS MALT lymphoma; (2) lesions located within the ventricular system; and (3) sufficient clinical and pathological data available. Exclusion criteria included: (1) secondary CNS involvement; (2) nonventricular lesions; and (3) review articles without individual case data.

## Discussion

In this case, histology revealed abundant choroid plexus tissue with marked stromal edema within the papillae, accompanied by extensive lymphoid tissue proliferation. The proliferating lymphoid tissue formed well-defined follicular structures within the choroid plexus papillae. IHC showed CD10- and Bcl-6-positive cells, indicating the presence of residual germinal centers. CD35 and CD21 highlighted the FDC meshwork. Tumor cells were negative for CD10 and Bcl-6, excluding follicular lymphoma. The expanded marginal zone cells appeared as small lymphocytes and monocytoid cells, expressing the B-cell markers CD79a and CD20, while the T-cell markers CD3 and CD5 were negative, supporting the diagnosis of a small B-cell lymphoma. Ki-67 demonstrated high proliferative activity within the germinal centers, whereas the hyperplastic marginal zone showed low proliferation. Cyclin D1 negativity excluded mantle cell lymphoma. Tumor cells were negative for CD5 and CD23, ruling out chronic lymphocytic leukemia/small lymphocytic lymphoma. The differential diagnosis also included DLBCL, reactive lymphoid hyperplasia, and IgG4-related disease. DLBCL is characterized by large, atypical lymphoid cells with high mitotic activity and a high Ki-67 proliferation index, whereas MALT lymphoma exhibits small- to medium-sized cells with monocytoid features and a lower proliferation index. Reactive lymphoid hyperplasia typically preserves the underlying tissue architecture, shows a mixed population of B and T cells, and demonstrates polyclonality on molecular studies, in contrast to the monoclonal B-cell population observed in MALT lymphoma. IgG4-related disease typically shows dense lymphoplasmacytic infiltration and fibrosis but usually lacks clonal B-cell expansion. It is often characterized by increased numbers of IgG4-positive plasma cells and elevated serum IgG4 levels. In the present case, negative IgG4 immunostaining together with a normal serum IgG4 level does not support a diagnosis of IgG4-related disease. Histologic and molecular features support a diagnosis of MALT lymphoma. Due to the retrospective nature of this study, several molecular analyses were not performed, including MYD88 L265P mutation, t(11;18)(q21;q21)/API2-MALT1 rearrangement, trisomy 3/18, BIRC3-MALT1 fusion, and EBER *in situ* hybridization, which represents a limitation of this study.

MALT lymphomas arising in the lateral ventricle share histological and immunohistological features with MALT lymphomas at other sites, composed of small lymphocytes, marginal zone cells, and with or without plasmacytic differentiation. The tumor cells showed abundant clear cytoplasm (monocytoid morphology). According to the updated WHO classification of CNS tumors, primary CNS MALT lymphoma is rare and almost exclusively arises in the dura, known as MALT lymphoma of the dura. Dural meningeal epithelial cells originate from mesothelial (13). The choroid plexus consists of invaginated folds of pia mater (vascular leptomeninges), covered by a single layer of modified ependymal cells. The choroid plexus originates from two distinct cell lineages: the mesectoderm, which also gives rise to pial and arachnoid cells (13), and the neuroectoderm, from which ependymal cells are derived (14). Although anatomically distinct, we propose that lateral ventricular MALT lymphoma may originate from the pia mater folds of the choroid plexus, resembling the dural origin of MALT lymphoma. This shared meningothelial-like cellular origin may account for their overlapping histological and immunophenotypic features and suggests similar pathogenetic pathways in CNS MALT lymphomagenesis. In addition, cerebrospinal fluid-derived marginal zone precursor cells may home to a chronic inflammatory microenvironment within the choroid plexus stroma, thereby contributing to the development and progression of the disease. However, these hypotheses remain unproven, and further studies are needed.

According to the currently published literature, a total of eight cases of MALT lymphoma arising in the ventricular system have been reported. The common symptoms are headache or seizures. The initial clinical diagnosis considered meningioma, metastasis, or ependymoma. The median age of patients is 53 years, which is consistent with that of dural MALT lymphoma, whose overall median age is approximately 60 years (15). Women are affected more often than men. Six cases underwent gross total resection, with or without adjuvant therapy. The longest follow-up period was 25 months, during which the patient showed no evidence of disease. MALT lymphoma outside the CNS has been attributed to chronic inflammation (16). In the CNS, it remains unclear whether MALT lymphoma in the lateral ventricle is associated with inflammatory diseases. One patient had a history of previous trauma and chronic inflammation, and another patient had *Chlamydia psittaci* infection (10, 12). In this case, the patient had no evidence of infection.

Here, we report a case of primary CNS MALT lymphoma arising in the lateral ventricle, an anatomical site lacking dural structures. Unlike dural MALT lymphoma, this unusual localization suggests that lateral ventricle MALT lymphoma may originate from the pia mater of the choroid plexus. Although arising from distinct anatomical sites, both dural and lateral ventricle MALT lymphomas may originate from meningeal-associated lymphoid tissue, which could account for their overlapping histological and immunophenotypic features. Although the precise etiology remains unclear, this observation expands the current WHO definition of CNS MALT lymphoma and raises the possibility of a pathogenic association with the meninges. Furthermore, distinguishing primary MALT lymphoma in the lateral ventricle from meningioma, metastasis, or ependymoma based on imaging alone remains challenging, and MALT lymphoma should be considered in the differential diagnosis of intraventricular tumors.

## Data Availability

The original contributions presented in the study are included in the article/supplementary material. Further inquiries can be directed to the corresponding authors.
